# Liquid Glass for Stiff Bandages in Fractures

**Published:** 1871-01

**Authors:** 


Liquid Glass for Stiff Bandages in Fractures.—Dr. John Darby, of South Carolina, lauds very highly liquid silicates for an immovable dressing; he regards it as superior to any of the substances heretofore in use, on account of its neatness, durability, strength, and ease of application.

He first envelopes the limb in wadding or cotton, putting a larger bandage over it; this is then saturated with the silicate, it being applied with a painter's brush. Another bandage is applied over this, and the liquid glass painted on again. Strips of muslin, pasteboard, felt, or veneering wood, soaked in the liquid, may be applied on different parts of the dressing, making the whole of any thickness and strength desirable.

After the dressing is completed, the limb must be kept perfectly motionless until the material hardens, which will require at least as many hours as there are layers of the bandage, and perhaps many more; from eight to twelve hours usually suffices.

In removing, the scissors or knife is used. Whenever a limb is required to be kept in a position of absolute immobility, he regards this a very superior dressing.—*Author's Pamphlet, Etc.*

